# Acute Liver Failure in Rats Activates Glutamine-Glutamate Cycle but Declines Antioxidant Enzymes to Induce Oxidative Stress in Cerebral Cortex and Cerebellum

**DOI:** 10.1371/journal.pone.0095855

**Published:** 2014-04-22

**Authors:** Santosh Singh, Papia Mondal, Surendra K. Trigun

**Affiliations:** 1 Department of Zoology, Guru Ghasidas Vishwavidyalaya, Bilaspur, Chhattisgarh, India; 2 Biochemistry and Molecular Biology Laboratory, Department of Zoology, Banaras Hindu University, Varanasi, Uttar Pradesh, India; Laurentian University, Canada

## Abstract

**Background and Purpose:**

Liver dysfunction led hyperammonemia (HA) causes a nervous system disorder; hepatic encephalopathy (HE). In the brain, ammonia induced glutamate-excitotoxicity and oxidative stress are considered to play important roles in the pathogenesis of HE. The brain ammonia metabolism and antioxidant enzymes constitute the main components of this mechanism; however, need to be defined in a suitable animal model. This study was aimed to examine this aspect in the rats with acute liver failure (ALF).

**Methods:**

ALF in the rats was induced by intraperitoneal administration of 300 mg thioacetamide/Kg. b.w up to 2 days. Glutamine synthetase (GS) and glutaminase (GA), the two brain ammonia metabolizing enzymes vis a vis ammonia and glutamate levels and profiles of all the antioxidant enzymes vis a vis oxidative stress markers were measured in the cerebral cortex and cerebellum of the control and the ALF rats.

**Results:**

The ALF rats showed significantly increased levels of ammonia in the blood (HA) but little changes in the cortex and cerebellum. This was consistent with the activation of the GS-GA cycle and static levels of glutamate in these brain regions. However, significantly increased levels of lipid peroxidation and protein carbonyl contents were consistent with the reduced levels of all the antioxidant enzymes in both the brain regions of these ALF rats.

**Conclusion:**

ALF activates the GS-GA cycle to metabolize excess ammonia and thereby, maintains static levels of ammonia and glutamate in the cerebral cortex and cerebellum. Moreover, ALF induces oxidative stress by reducing the levels of all the antioxidant enzymes which is likely to play important role, independent of glutamate levels, in the pathogenesis of acute HE.

## Introduction

Hepatic encephalopathy (HE) is a neurological disorder found in patients with acute and chronic liver failure. On the basis of the underlying hepatic abnormalities, three types of HE have been categorized, type A: encephalopathy associated with the acute liver failure (ALF), type B: associated with the portal-systemic bypass but with no intrinsic hepatocellular disease and type C: associated with the cirrhosis and portal hypertension/or portal-systemic shunts [1]. There is a general agreement that increasing concentration of ammonia in the brain acts as the main pathogenic factor for all types of HE [2]. The increased brain ammonia is speculated to create imbalance of glutamate and gama amino butyric acid (GABA) neurotransmitter functions and to produce neuropsychiatric abnormalities in the HE patients [3], [4].

At downstream to imbalanced neurotransmission functions, oxidative stress is speculated to play major roles in cerebral ammonia toxicity and thus, ammonia-glutamate-excitotoxicity-oxidative stress pathway has been focused much to understand HE pathogenesis [3], [5]. It has been proposed that increased ammonia level in the brain initially affects astrocytes biochemistry followed by inducing oxidative stress not only in the astrocytes but in the neurons also [6]. The increased production of reactive oxygen and nitrogen oxide species (ROS/RNOS) in the brain cells, in turn, is likely to amplify the neuronal derangements associated with the HE pathogenesis [6]. We have also reported that elevated brain ammonia level, in a pure hyperammonemic (with normal liver function) rat model correlates well with the increased oxidative stress vis a vis declined antioxidant enzymes in cerebral cortex and cerebellum of those rats [7]. Moreover, in most of these studies, the role of alterations in brain ammonia metabolism has been ignored while deriving a correlation between brain ammonia *vis a vis* glutamate levels, neuro-excitotoxicity and oxidative stress [8].

Normally, ammonia is produced in small intestine by the colonic bacteria and by deamination of glutamine [5]. After absorption, ammonia follows a first-pass effect to reach the liver where it is detoxified by the urea cycle enzymes to produce urea. Due to compromised urea cycle during liver failure condition, ammonia level gets elevated in the blood causing hyperammonemia (HA) [3]. Since, ammonia crosses the blood brain barrier easily; it reaches the brain without hindrance. There is no effective urea cycle in the brain. However, brain cells operate glutamine-glutamate cycle which is catalyzed by glutamine synthetase (GS) and glutaminase (GA) found in the astrocytes and neurons respectively [9]. The glutamine produced by the astrocytic GS enters into the adjacent neurons where it is converted into glutamate by GA [10]. It is argued that during liver failure, this cycle in the brain gets overloaded resulting into excessive ammonia/glutamate trafficking. This may result into astrocytes swelling, characterized as Alzheimer Type II astrocytosis, which is considered as one of the hall marks of the chronic type HE [3], [11]. There are some reports on decline of GS level in the astrocytes exposed to HA *in vitro* and in the brain of the animals administered with high concentration of the ammonium salts [3], [6], [12]. Though some findings independently suggest alterations in GA levels and oxidative stress markers in the brain during chronic HE [13], [14], the association between GS-GA cycle and oxidative stress in the brain of a true liver failure HE model remains undefined.

The brain is one of the most vulnerable organs with regard to the oxidative stress because of its high oxygen demand and abundance of polyunsaturated fatty acid enriched myelinated nerve fibers [15]. To get rid of ROS insult, the brain has robust network of antioxidant defense system wherein, antioxidant enzymes play important roles. Amongst them, superoxide dismutase (SOD) and catalase scavenge O_2_^•−^ to produce H_2_O and O_2_. On the other hand, SOD, glutathione peroxidase (GPx) and glutathione reductase (GR) channel O_2_^•−^ in a nicotinamide adenine dinucleotide phosphate (NADPH) dependent pathway to maintain reduced glutathione (GSH)/oxidized glutathione (GSSG) ratio. Together, the synchronized activities of all these antioxidant enzymes play important roles in protecting brain cells from the oxidative damage [7].

We have reported that acute HA led increased ammonia in the brain declines most of the antioxidant enzymes and thereby induces oxidative stress in the hyperammonemic brain [7]. The oxidative stress of similar intensity could be observed in the brain of the thioacetamide (TAA) induced true ALF rats also, however, with static levels of ammonia and glutamate (data of the present article). This hinted towards induction of oxidative stress even when brain is able to prevent glutamate elevation during ALF. In order to understand biochemical mechanism of such cerebral changes, we have investigated the concordant profiles of ammonia metabolizing enzymes (GS and GA) vis a vis ammonia and glutamate levels and antioxidant enzymes vis a vis ROS level in cerebral cortex and cerebellum of the TAA induced ALF rats.

## Materials and Methods

### Animals and Chemicals

Adult male albino rats weighing 200–220 g were used in this study. Rats were fed with the recommended diet and maintained at standard conditions in an animal house. The use of animals for the present study was approved by the institutional animal care and use committee (IACUC); Animal ethical committee of the Banaras Hindu University, Varanasi (Ref. No. Dean/2006/07/805).

Chemicals used were of analytical grade supplied by the E-Merck, SRL and Glaxo (INDIA). Acrylamide, *N,N’*-methylene bis acrylamide, Coomassie Brilliant Blue R-250 (CBB), N,N,N’N’-tetramethylethylenediamine (TEMED), phenyl methyl sulphonyl fluoride (PMSF), Ammonia, glutamate and glutamine assay kits were purchased from Sigma-Aldrich, USA. GOT (glutamate oxalate transaminase) and GPT (glutamate pyruvate transaminase) assay kits were obtained from Span Diagnostics Ltd., India.

### Induction of Acute Liver Failure

ALF in rats was induced by intraperitoneal administration of 300 mg TAA/Kg b.w. prepared in 0.9% NaCl, to 5–7 rats once daily up to two days as described earlier [16]. Control rats were administered with the same volume of 0.9% NaCl. To minimize weight loss, hypoglycemia and renal failure in the experimental rats, 5% dextrose solution containing 0.45% NaCl and 20 meq/L potassium chloride was given through drinking water as supportive therapy to all the rats [17].

### Liver Histology

Liver histology was performed as described earlier from our lab [17]. In brief, the liver tissue from control and experimental rats was excised, sliced into 0.3–0.5 cm blocks and fixed in Bouin’s solution for 18–24 h. Tissue blocks were transferred to 70% ethanol, processed for alcoholic dehydration and embedded in paraffin. Liver sections (6 µm) were cut followed by hematoxylin - eosin (H-E) staining and examined under microscope.

### Preparation of Tissue Extracts

Mitochondria free cerebral cortex and cerebellar extracts were prepared in an extraction medium consisted of 400 mM sucrose, 1 mM EDTA, 0.2 mM benzamidine, 0.1 mM PMSF (pH 7.4) and 0.02% heparin. Extracts were centrifuged at 12,000 g for 15 min and then at 19,000 g for 30 min at 4°C to obtain cytosolic fractions.

### Biochemical Estimations

As described previously [4], [16], sGOT (EC 2.6.1.1), sGPT (EC 2.6.1.2), ammonia and glutamate were assayed in the serum from the pooled blood from 4–5 rats and/or in the brain tissue from each group following the procedure mentioned in the kits supplied by Sigma-Aldrich, USA. Protein content was determined by Lowry method.

Serum profiles of lactate dehydrogenase (LDH; EC1.1.1.27) isozymes were studied using non-denaturing polyacrylamide gel electrophoresis (native PAGE). Serum containing 10 µg protein was loaded in each lane of 7.5% gel and electrophoresis was performed under non denaturing conditions at 4±2°C. The gels, after electrophoresis, were stained in LDH activity staining mixture as reported earlier [17].

Level of malondialdehyde (MDA), the product of lipid peroxidation, was determined as described previously [7]. The total glutathione (GSH+GSSG) was measured following the method of Sedlak and Raymond [18].

The protein carbonyl content was determined as describe previously [19]. Briefly, sample was incubated with streptomycin sulfate solution (1%) for 15 min. The mixture was centrifuged at 3600 g. The supernatant obtained was incubated with either 10 mM DNPH in 2 M HCl or with only 2 M HCl (blank) for 1 h at room temperature. Protein was precipitated by adding an equal volume of 20% TCA. After centrifugation (8600 g), the pellet was washed thrice with ethanol: ethyl acetate (1∶1) mixture for removing excesses of DNPH. The precipitate was re-dissolved in 6 M guanidine HCl and O.D. was recorded at 370 nm. Carbonyl content was calculated using (ε) = 22 mM^−1^ cm^−1^ mg^−1^ protein.

### GS

GS (EC 6.3.1.2.) activity was measured by monitoring the γ-glutamyl transferase reaction as described previously [20] with some modifications. Briefly, the reaction mixture contained 20 mM imidazole-HCl buffer (pH 7.2), 50 mM L-glutamine, 60 mM hydroxylamine-HCl (pH 7.0), 2 mM MnCl_2_, 20 mM Na-arsenate, and 0.4 mM ADP in a final volume of 2 mL. The µmoles of γ-glutamyl hydroxamate formed after 15 min at 35°C was determined by recording A_540_ after addition of 0.5 mL solution containing 12% TCA, 6.7% FeCl_3_ and 0.5 N HCl. A standard curve was constructed against different concentrations of γ-glutamyl hydroxamate. One unit of the enzyme was defined as 1 µmole of γ-glutamyl hydroxamate/min.

### GA

GA (EC 3.5.1.2) catalyzes conversion of glutamine into glutamate. For assay of GA, this reaction was coupled with glutamate dehydrogenase (GDH) catalyzed production of 2-oxoglutarate following the method reported by Jeon *et al* [21].

The initial solution (0.1 mL of 2% L- glutamine and 0.2 mL of 100 mM Tris - HCl buffer; pH 7.5) was pre-incubated at 37°C for 5 min. The reaction was started by addition of 0.1 mL tissue extract and continued up to 10 min at 37°C. To stop the reaction, reaction mixture was boiled for 3 min and centrifuged at 8,000 g for 5 min. An aliquot of 50 µL from the supernatant was added to a reaction mixture containing 1 mL of hydroxylamine buffer (0.25 M hydroxylamine and 20 mM EDTA, pH 8.0), 0.5 mL 10 mM NAD^+^, 1 mL distilled water and 20 U GDH. After 30 min incubation at 37°C, O.D. was recorded at 340 nm. One unit of GA was defined as 1 µmol NADH produced/min at 37°C.

### Assay of SOD, Catalase, GPx and GR

As reported earlier [7], [17], SOD (EC: 1.15.1.1) activity was measured by recording inhibition of nitroblue tetrazolium (NBT) reduction in a NADH-Phenazonium Methosulphate (PMS)-NBT reaction system. The unit of SOD was defined as 50% inhibition of NBT reduction/min.

Catalase (EC: 1.11.1.6) was assayed by using a reaction mixture containing 0.01 M potassium phosphate buffer (pH 7.0) and 0.1 mL tissue extract [7]. Reaction was started by addition of 0.8 M H_2_O_2_ and stopped after 60 s by 2 mL dichromate acetic acid reagent. All the tubes were incubated in a boiling water bath for 10 min. The tubes were brought to the room temperature and absorbance was recorded at 570 nm. After comparing the data with a standard plot, constructed using a range of 10 - 160 µmoles of H_2_O_2,_ catalase activity was expressed as µmoles of H_2_O_2_ consumed/min/mg protein.

GPx (EC:1.11.1.9) activity was determined by monitoring the GR coupled reaction pathway wherein, GSSG, produced by GPx using GSH, is reduced back to GSH by coupling with NADPH oxidation in the presence of GR. Thus, the rate of NADPH oxidation was monitored at 340 nm (Є 6.22 mM^−1^⋅cm^−1^) in a reaction mixture consisted of 0.3 mM NADPH, 0.17 mM GSH, 50 mM phosphate buffer (pH 7.3), 0.5 mM H_2_O_2_, 20 µM sodium azide and 0.2 unit/mL GR [17]. Reaction was started by addition of the cytosolic fractions containing 0.3–0.5 mg protein. The unit of the enzyme was defined as nmol NADPH oxidized/min.

The reaction mixture used for the assay of GR (EC: 1.6.4.2) was same as reported previously [7], [17] and unit of the enzyme was defined as nmol NADPH oxidized/min.

### Native PAGE for SOD, Catalase, GPx and GR

SOD was separated by electrophoresis of the extracts containing 60 µg protein on 12% native PAGE. After electrophoresis, gels were incubated with 2.3 mM NBT, 28 µM riboflavin and 28 mM TEMED for 20 min in dark. After exposure of the gels under fluorescent light, achromatic SOD bands appeared against a dark blue background.

For catalase, tissue extracts containing 60 µg protein were electrophoresed on 8% PAGE. Gels were soaked for 10 min in 0.003% H_2_O_2_ and then incubated in a staining mixture consisting of 2% potassium ferricyanide and 2% ferric chloride. Achromatic catalase bands appeared against a blue-green background.

For GPx, after electrophoresis of 60 µg protein on 10% native PAGE, gels were incubated in 50 mM Tris-HCl buffer (pH 7.9) containing 13 mM GSH and 0.004% H_2_O_2_ for 20 min_._ Gels were finally stained with 1.2 mM NBT and 1.6 mM PMS. Achromatic GPx bands appeared against a violet–blue background.

The level of active GR was determined following the method of Wen-Chi Hou *et al*. [22] with some modifications. After 10% native PAGE of 60 µg protein in each lane, gels were incubated with 50 mM Tris-HCl (pH 7.9) containing 4 mM GSSG, 1.5 mM NADPH and 2 mM 5,5′-dithiobis (2-nitrobenzoic acid) (DTNB) for 20 min. Gels were stained in a GR activity stain mixture containing 1.2 mM NBT and 1.6 mM PMS for 10 min in dark. The achromatic active GR bands appeared against a purple-bluish background.

The intensity of bands in each case was quantified by gel densitometry using alphaimager 2200 gel documentation software. Specificity of the PAGE analyzed enzymatic bands was confirmed by obtaining a negative result with the similarly run gels stained in the corresponding activity stain mixtures but minus substrates of the concerned enzymes.

### Statistical Analysis

For two group comparisons, unpaired Student's t test was performed. The data have been expressed as mean ± SD. The probability of p<0.05 was taken as significant difference between the two groups.

## Results

### Induction of Acute Liver Failure

**Figure 1:**
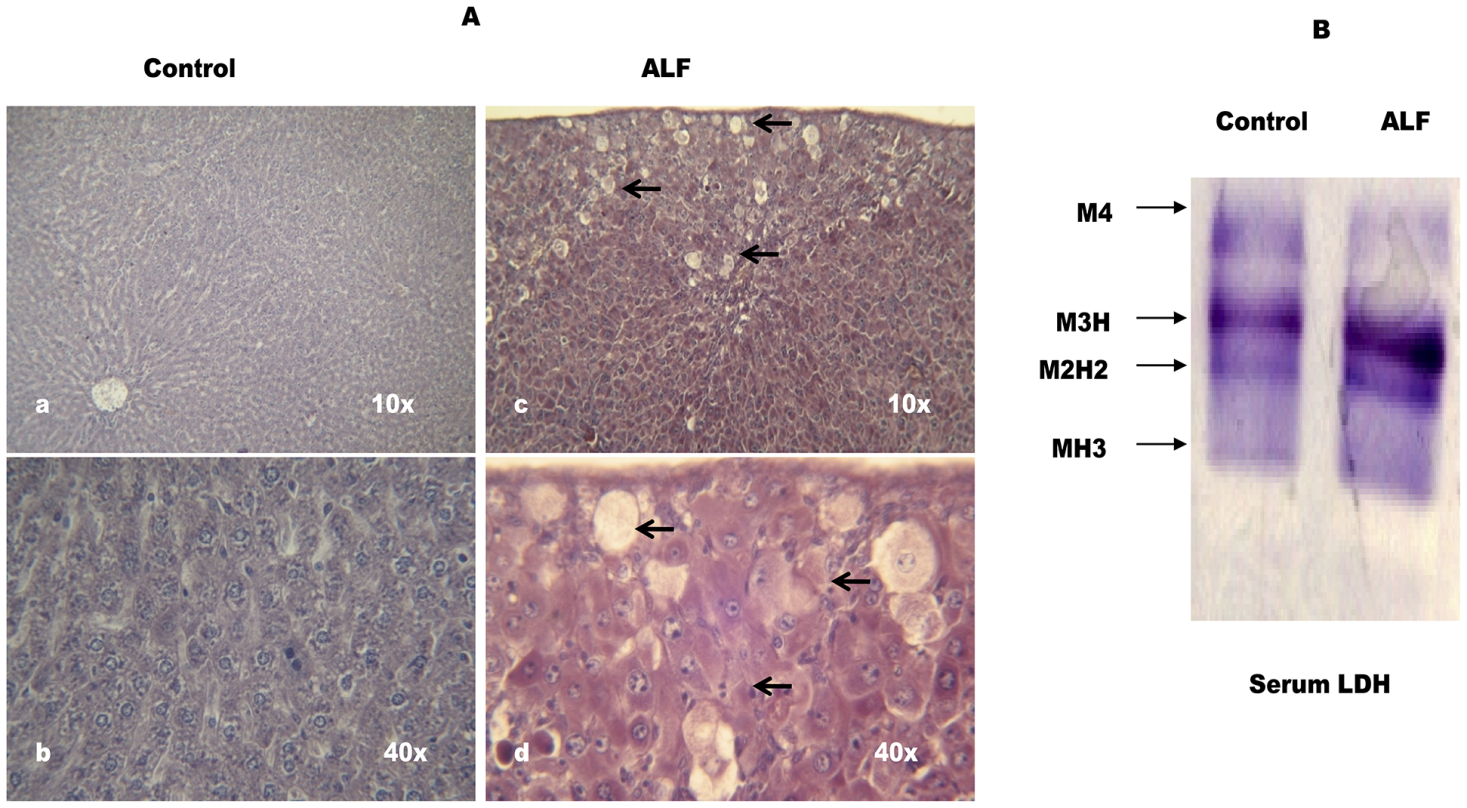
TAA induces severe hepatic cell damage in the rats. (A) Light micrographs of hematoxylin - eosin stained liver sections from control (a and b) and TAA treated (c and d) rats wherein, a and c are photomicrographs at 10× and b and d at 40× magnifications. Arrow heads show centrilobular necrosis in the liver cells. Cells with small nuclear stains indicate infiltration of neutrophils and mononuclear lymphocytes. In B, pooled serum from 4–5 rats containing 10 µg protein in each lane was electrophoresed on 7.5% non-denaturing PAGE followed by substrate specific development of LDH bands. The gel photograph is a representative of 4 PAGE repeats.

TAA is considered as an appropriate hepatotoxin to produce liver failure model of HE [23]. Based on the pilot experiments, we used 300 mg/Kg b.w of TAA to induce ALF in the rats. According to Fig-1A, many hepatocytes show classical centrilobular necrosis with infiltration of neutrophils and mononuclear lymphocytes in liver section of the TAA treated rats. Serum LDH profile is an important marker to ascertain *in* *vivo* tissue damage. Liver expresses M subunit dominating LDH isozymes (M4, M3H, M2H2). Fig-1B shows significantly increased levels of M3H and M2H2- LDH in serum of the TAA treated rats (Fig-1B) and thus, further confirmed significant liver cell damage in these rats.

The increased levels of sGOT and sGPT are used as markers to ascertain different grades of liver failure. As compared to the control rats, ∼14–15 fold increase in sGOT and ∼ 3–4 fold increase in sGPT were observed in serum of the TAA treated rats (Table-1).

The metabolic insufficiency of hepatic cells, with respect to mobilization of ammonia, was confirmed by a significantly increased ammonia level (∼ 2.3 fold; p<0.01) in serum of the TAA treated rats (Table-1). Taking together, these results suggested TAA induced acute liver failure in the rats showing enhanced serum ammonia concentration similar to that found in the acute type HE patients.

### Neurochemical Studies in Cerebral Cortex and Cerebellum of ALF Rats

#### Level of ammonia and glutamate

Ammonia and glutamate levels were observed to be unchanged in both, cerebral cortex and cerebellum of the ALF rats (Table-2).

#### Activities of GS and GA

**Figure 2:**
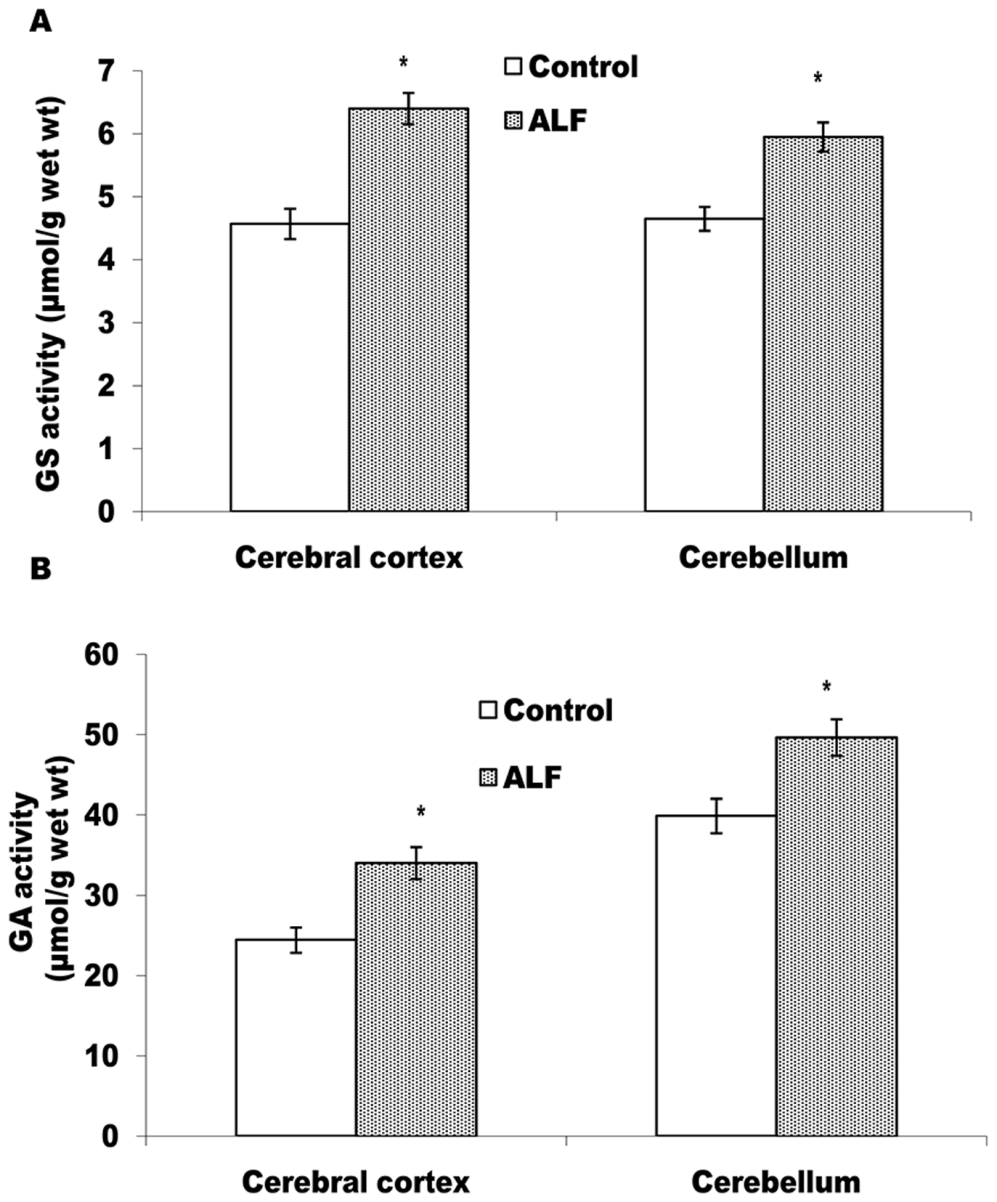
GS and GA get activated in cerebral cortex and cerebellum of the ALF rats. Activity profiles of GS (A) and GA (B) from control and ALF rats. The values represent mean ± SD where n = 4 *p<0.05 (control vs ALF rats).

GS and GA regulate ammonia metabolism in the brain cells. According to Fig-2, as compared to the control rats, GS (Fig-2A) and GA (Fig-2B) activities are increased significantly (p<0.05) in both, cerebral cortex and cerebellum of the ALF rats and thereby, suggesting activation of GS-GA cycle in brain of the ALF rats.

#### Level of oxidative stress parameters

Measuring MDA level (a stable product of lipid peroxidation) and protein carbonyl content (marker of protein oxidation) are the two useful tools for measuring oxidative damage at cellular level. According to Table-3, as compared to the control rats, the ALF rats showed significant increase in the MDA level in cerebral cortex (1.8 fold; p<0.05) and cerebellum (1.4 fold; p<0.05). Similarly, protein carbonyl content was increased ∼1.7 fold in cerebral cortex and ∼1.4 fold in the cerebellum. The level of total GSH, as a measure of reducing equivalents in the brain cells, is observed to be increased significantly in cerebral cortex, however, with a static pattern in cerebellum of the ALF rats.

#### Effect of ALF on the antioxidant enzymes

**Figure 3:**
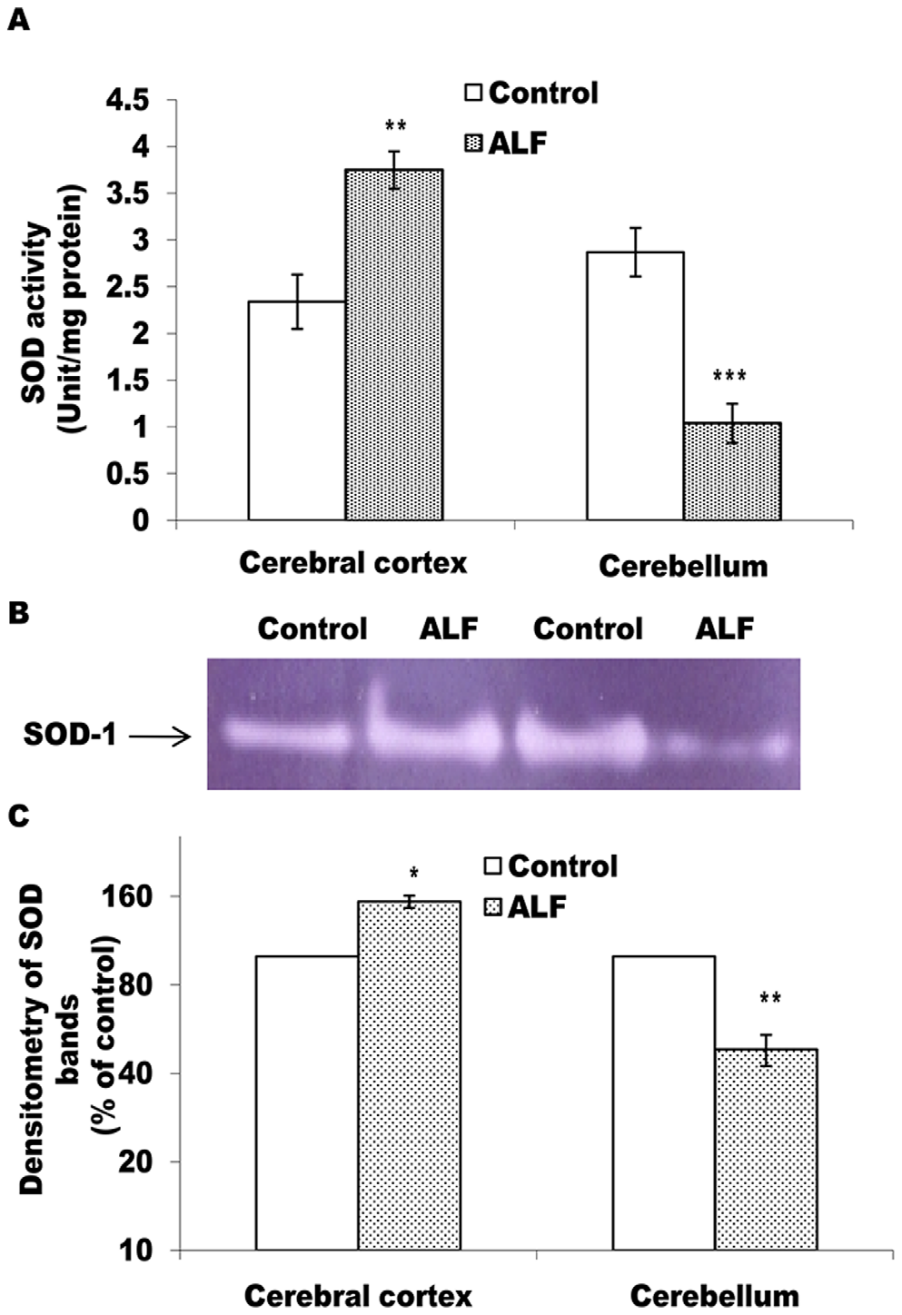
SOD activity shows differential pattern in cerebral cortex and cerebellum of the ALF rats. A represents SOD activity measured in cell free extracts; values are mean ± SD where n = 4 and each experiment done twice. In case of B, pooled tissue extracts from 4 rats containing 60 µg protein in each lane was electrophoresed on 12% non-denaturing PAGE followed by substrate specific development of SOD bands. The gel photograph in B is a representative of 4 PAGE repeats. In panel C, relative densitometric values of SOD band from experimental group, as % of the control lane, have been presented as mean ± SD from 4 PAGE repeats. *p<0.05; **p<0.01, ***p<0.001 (control vs ALF rats).

In mammalian cells, O_2_^•-^ is neutralized/metabolized by the synchronized activities of SOD and catalase and/or by SOD and GPx. As compared to the control group rats, the ALF rats showed significantly (p<0.05) increased level of active SOD in the cortex but a significant decline (p<0.001) in the cerebellum (Fig-3A). The intensity of SOD bands in gel also followed a similar trend in the cortex and cerebellum of the ALF rats (Fig-3B, C).

**Figure 4:**
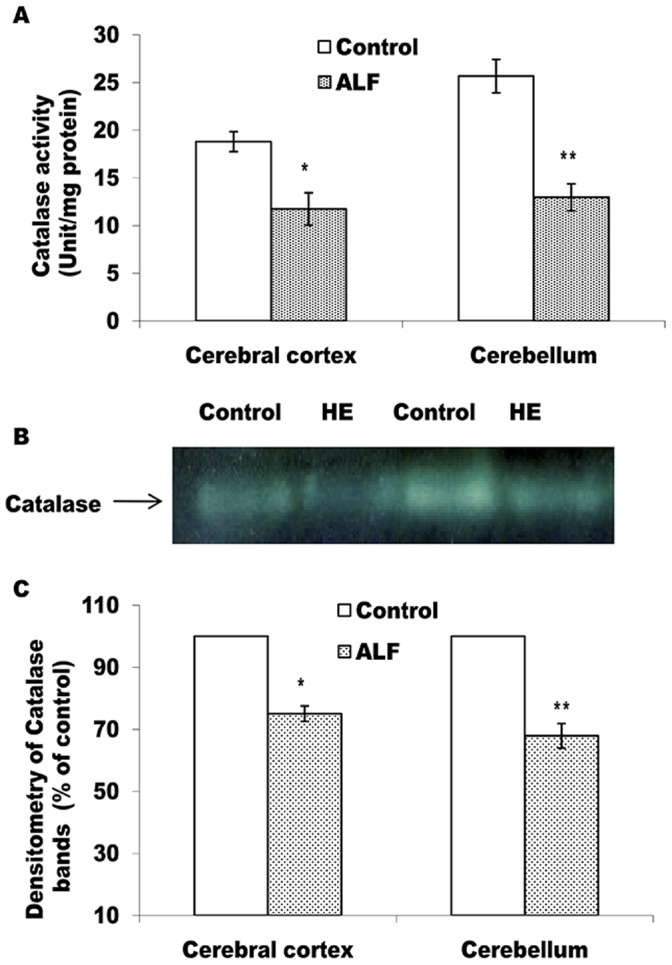
Level of active catalase declines in cerebral cortex and cerebellum of the ALF rats. Catalase activity measured in cell free extract (A) and non-denaturing PAGE pattern of active catalase (B and C). The technical details are same as described in Fig. 3 except 8% PAGE was used in case of B. *p<0.05; **p<0.01 (control vs ALF rats).

**Figure 5:**
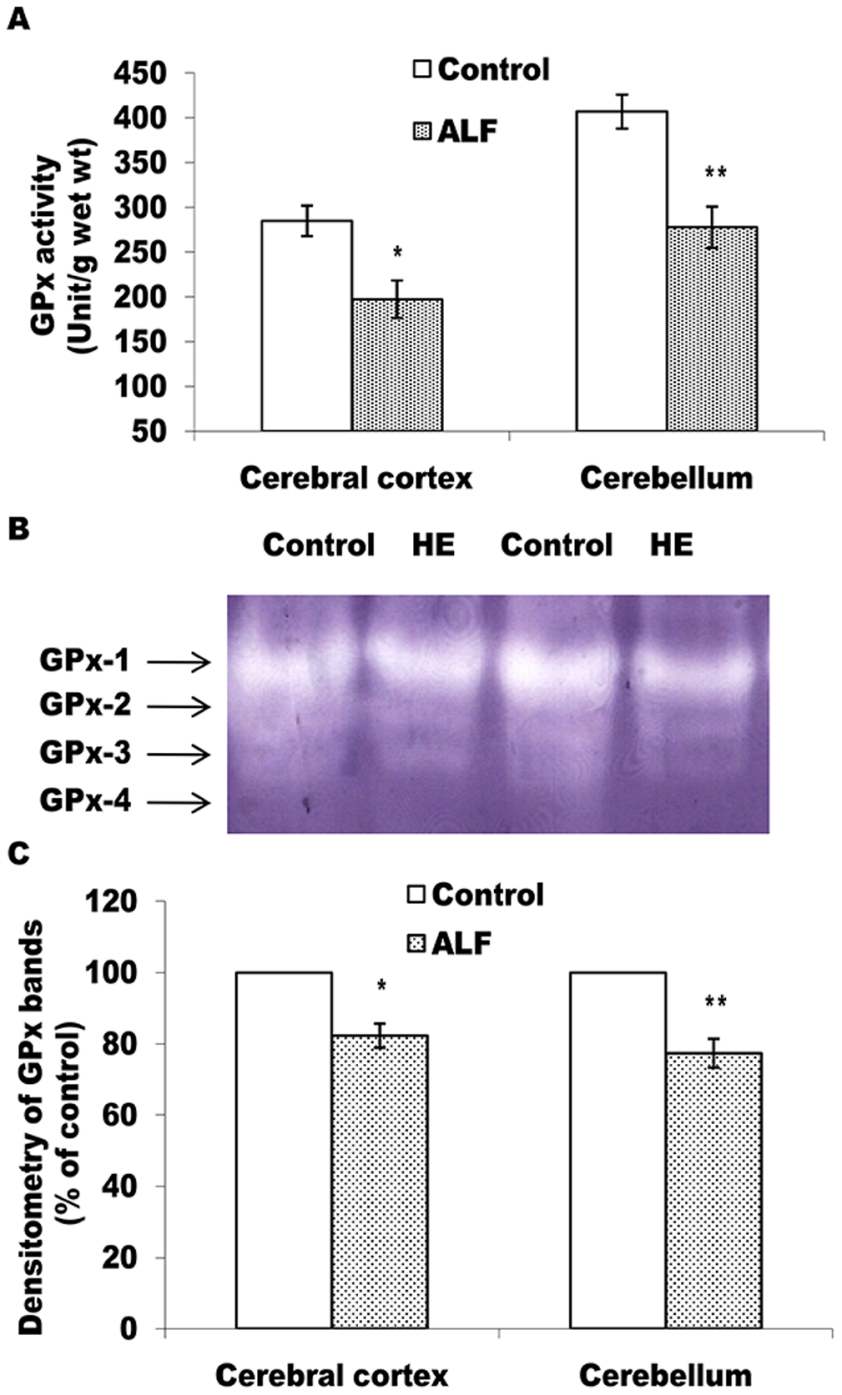
Level of active GPx declines in cerebral cortex and cerebellum of the ALF rats. GPx activity measured in cell free extract (A) and non-denaturing PAGE pattern of active GPx (B and C). The technical details are same as described in Fig. 3 except 10% PAGE was used in case of B. *p<0.05; **p<0.01 (control vs ALF rats).

**Figure 6:**
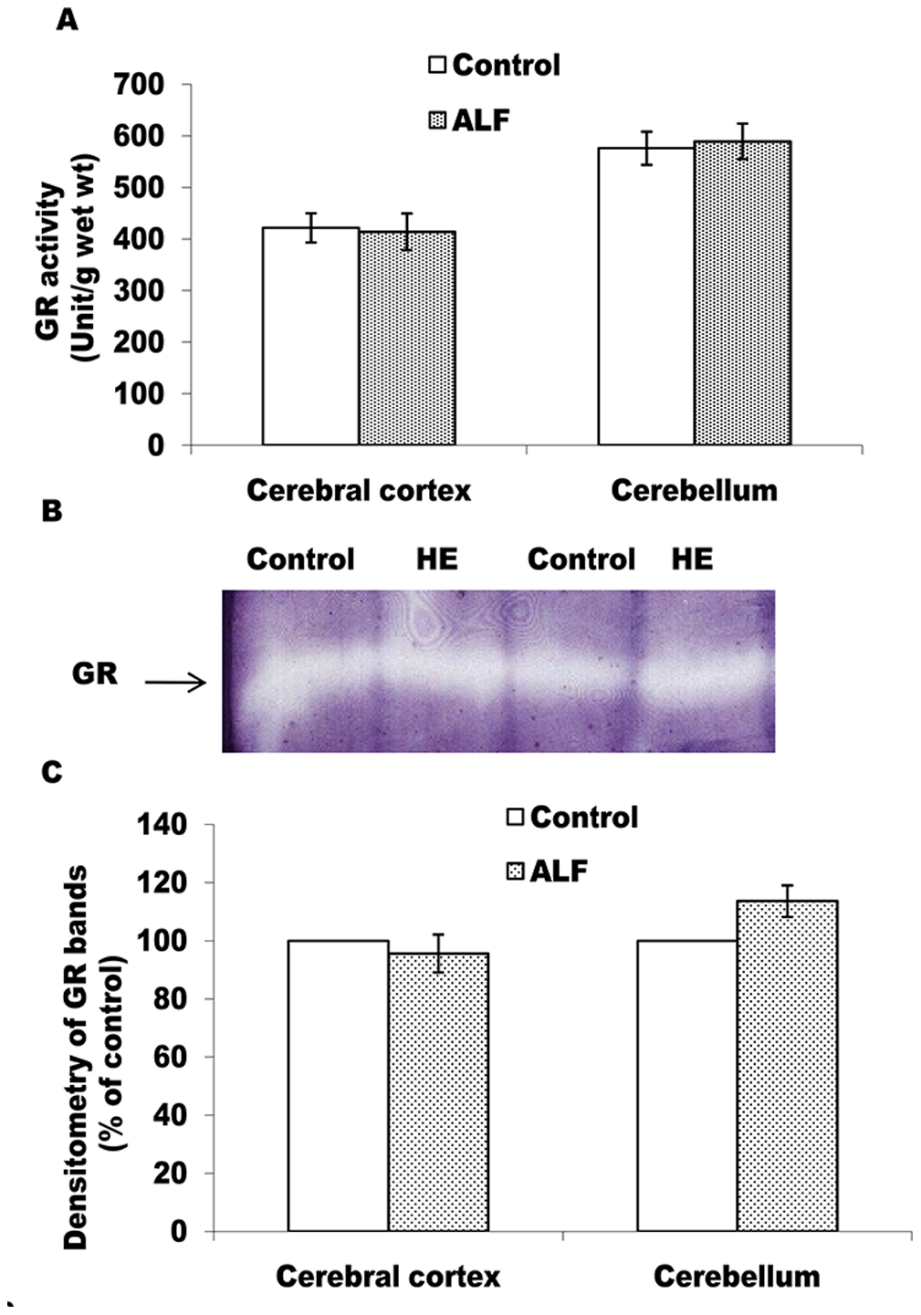
ALF did not induce alterations in the active level of GR in cerebral cortex and cerebellum. GR activity measured in cell free extract (A) and non-denaturing PAGE pattern of active GR (B and C). The technical details are same as described in Fig. 5.

According to Figs-4 and -5, as compared to the control rats, catalase and GP_X_ activities are found to be decreased significantly in both, the cerebral cortex and the cerebellum (Figs- 4A and 5A). Moreover, as compared to the cerebral cortex (p<0.05), cerebellum showed greater decline (p<0.01) in activities of both these enzymes. The intensity of catalase and all the four GPx bands in gel also showed similar declining trends in case of the ALF rats (Figs-4B, C and 5B, C). Contrary to this, in comparison to the control rats, GR activity (Fig-6A) and intensity of the GR bands (Fig-6B, C) remained unchanged in both the brain regions of the ALF rats.

## Discussion

Our previous report, that acute hyperammonemia in rats (without ALF) induces oxidative stress in cerebral cortex and cerebellum [7], led us to investigate the status of ammonia metabolism vis a vis oxidative stress in cerebral cortex and cerebellum of the ALF rats.

For understanding the pathogenesis of HE, the TAA induced ALF model has received much emphasis and acceptability by the International Society on Hepatic Encephalopathy and Nitrogen metabolism (ISHEN) because, it produces a condition of liver failure similar to that found in the cirrhotic patients. TAA is a selective hepatotoxin that causes hepatic cell necrosis and thereby produces liver dysfunction in a dose dependent manner [16], [17]. Therefore, based on the pilot experiments data, we used two doses of 300 mg TAA/Kg b.w and as reported earlier [17], performed conventional liver function tests (LFT) and histopathological studies to characterize ALF in those rats.

Differential pattern of LDH isozymes in the serum serves as a marker for the *in vivo* tissue damage. Liver expresses mainly M-subunit dominating LDH isozymes [17], [24]. Out of them, the enhanced serum levels of M3H and M2H2 have been reported to be closely associated with the TAA induced liver damage in the rats [17]. In the present context also, these two LDH isozymes are found to be increased significantly in the serum of the experimental rats (Fig. 1B) and thus indicating mainly liver damage in the TAA treated rats. Apparently no change in the serum levels of heart specific H-subunit dominating MH3-LDH (Fig-1B) provides further support to this argument. Moreover, increased levels of sGOT and sGPT are considered as standard LFT markers to determine the degree of liver dysfunction in the patients. We observed ∼ 14-fold increase in sGOT and ∼3-fold increase in sGPT levels in case of the TAA treated rats (Table 1). Since these values are close to the sGOT and sGPT levels reported in case of the fulminate hepatic rats induced by a similar dose of TAA [16], [25], these rats were considered to represent a true ALF model. A greater extent of hepatocytes necrosis seen in the liver sections from the TAA treated rats (Fig- 1A) also supported this conclusion. Similar histopathological changes have also been found associated with the acute type HE in the rats due to TAA treatment [16], [25]. Furthermore, ∼ 5-fold increase in serum ammonia concentration in the TAA-group rats (Table 1) correlates well with a similar HA condition reported for the ALF rats [16], [25]. Taking together, data in Fig-1 and Table-1 clearly suggested induction of ALF with the acute type HA condition in these rats and thus, they were considered suitable to discern neurochemical inferences with respect to the acute type HE.

**Table 1:** Changes in LFT markers and ammonia in serum of the ALF rats.

Parameters	Control rats	ALF rats
sGOT (IU/L)	550±27	7500±31 **
sGPT (IU/L)	1000±63	3300±87 **
Ammonia (µmol/ml)	0.29±0.03	1.45±0.04***

The data represents Mean ± S.D, where n = 4, **p<0.01, ***p<0.001 (control vs ALF group).

Ammonia easily crosses the blood brain barrier [26] and therefore, extent of the ammonia induced neuronal derangements has a direct bearing on status of the ammonia metabolism in the brain. As reviewed previously [4], in the brain, ammonia is metabolized mainly by the synchronized activities of astrocytic GS and neuronal GA. The GS catalyzes incorporation of ammonia into glutamate to synthesize glutamine in the astrocytes. Astrocytic glutamine is then transported to the pre-synaptic neurons where it is deaminated by GA to produce an excitatory neurotransmitter glutamate [10]. Thus, profiles of GS and GA vs glutamate-glutamine cycle become critical in imposing ammonia induced neurological complications during ALF/acute HA condition [27]. There is a general agreement that acute HA causes enhanced glutamate production and in turn glutamate excitotoxicity in the post-synaptic neurons [3]. However, reports are limited and also inconsistent on the profiles of the enzymes responsible to maintain high glutamate turnover in the brain during acute HA/HE. Some findings on *in vitro* and *in vivo* HA models have suggested that acute HA causes decline in both, the GS activity and the astrocytic glutamate transporters resulting into enhanced glutamate level in the brain [4]. There is a report on the increased GA activity in brain of the rats with portacaval anastomosis [13]. Also, neuro-pathogenesis of ammonia has been reported to show differential pattern in different brain regions [3], [7], [16], [17]. Since, cerebral cortex and cerebellum are considered to be affected more during HA [28] and both these brain regions have been shown to respond differently with respect to certain HA induced neurochemical changes [7], we attempted to study the profiles of GS and GA vs ammonia and glutamate levels concordantly in both these brain regions of the ALF rats.

We observed a significant increase in blood ammonia level (Table-1) but a static pattern of ammonia concentration in both, cereberal cortex and cerebellum of the ALF rats (Table-2). This suggested efficient metabolism of excess ammonia in both the brain regions of the ALF rats. The significant increases in GS and GA activities in these brain regions (Fig-2) provide support to this argument. Similarly, activation of glutamine-glutamate cycle, due to concordant increase in both these enzymes, could be held accountable for maintaining static level of glutamate observed in both the brain regions of the ALF rats (Table-2). Though these findings differ from some reports describing HA induced inhibition of GS activity and increased glutamate level in the brain [4], in the present context, they provide an enzymatic mechanism to suggest efficient ammonia metabolism in cerebral cortex and cerebellum of the ALF rats. The brain cells exhibit a greater degree of metabolic plasticity during un-physiological challenges [29] including HA induced enzymatic changes for maintaining alternate energy substrates [30]. HA exposed brain slices have also been shown to modulate most of the antioxidant enzymes to protect the brain cells from oxidative damage [31]. Thus, it is likely that cerebral cortex and cerebellar cells protect themselves from ammonia neurotoxicity by activating GS-GA cycle during TAA induced ALF condition.

**Table 2:** Levels of ammonia and glutamate in cerebral cortex and cerebellum of the control and ALF rats.

Brain region	Biochemical parameters	Control rats	ALF rats
Cerebral Cortex	Ammonia (mM/g)	1.21±0.07	1.10±0.05
	Glutamate (µM/g)	2.67±0.09	2.79±0.10
Cerebellum	Ammonia (mM/g)	1.45±0.012	1.32±0.07
	Glutamate (µM/g)	2.55±0.05	2.89±0.08

The data represents Mean ± S.D, where n = 4.

It has been described that increased ammonia in the brain induces glutamate dependent N-methyl-D-aspartate receptor (NMDAR) activity which, in turn, may lead into enhanced ROS production in the brain cells [3]. Though we did not observe increase in ammonia and glutamate levels in cerebral cortex and cerebellum of the ALF rats, it was interesting to observe significantly increased levels of lipid peroxidation and protein carbonyl contents in both these brain regions of the ALF rats (Table-3). This hinted towards ALF induced oxidative damage in both the brain regions even when brain ammonia and glutamate levels are maintained within the normal range.

**Table 3:** Changes in the levels of oxidative stress markers in cerebral cortex and cerebellum of the ALF rats.

Brain region	Biochemical parameters	Control rats	ALF rats
Cerebral Cortex	Lipid peroxidation (MDA nmol/g)	55.60±3.08	96.80±4.73**
	Protein carbonyl content (µmol/g)	48.63±4.55	82.18±4.63**
	Total glutathione (GSH+GSSG) (nmol/mg protein)	14.89±1.1	18.21±1.56*
Cerebellum	Lipid peroxidation (MDA nmol/g)	63.96±3.05	88.48±2.12*
	Protein carbonyl content (µmol/g)	49.55±5.92	65.51±5.13*
	Total glutathione (GSH+GSSG)(nmol/mg protein)	12.38±1.12	14.82±1.67

The data presents Mean ± S.D, where n = 4, *p<0.05, **p<0.01, (control vs ALF group).

There could be two mechanisms to explain this mismatch. First, it has been postulated that increased level of ammonia causes accumulation of glutamine in the astrocytes to produce Alzheimer’s type II astrocytosis [3]. Since, we did not observe such cellular changes in the brain of ALF rats (unpublished result), it is speculated that the amount of ammonia reaching to the brain of these rats is not sufficient enough to cause astrocytosis. It has been reported that such astrocytic changes are observed more during chronic HE than that of the acute type end stage HE [32]. Nonetheless, enhanced entry of ammonia in brain is known to increase glutamine concentration in the astrocytes and thereby, triggers ROS production in the brain cells [6], [27]. A similar condition may exist in the present context also, where high influx of ammonia in the brain could initially enhance glutamine level in the astrocytes to get rid of high ammonia load and in turn, could provoke ROS production in these brain regions of the ALF rats.

The second possibility could be the over activation of NMDAR at the post synaptic neurons during ALF [3] resulting into excess ROS production. There is a strong neurochemical basis to support this argument. A direct molecular link exists between NMDAR, post synaptic density protein-95 (PSD-95) and neuronal nitric oxide synthase (nNOS) to enhance nitric oxide (NO) production in the post synaptic neurons upon glutamate-NMDAR activation [33]. The NO produced so in the post synaptic neurons is known to increase ROS production by uncoupling mitochondrial oxidative phosphorylation in the brain cells [16], [34]. Moreover, to make it happen, excess of glutamate is supposed to be maintained in the synapses [3], [4], [35]. In the present context, therefore, it may be argued that though glutamate level in cerebral cortex and cerebellum of the ALF rats remain static but due to the activation of GS-GA cycle, it is likely to be maintained for a longer duration resulting into sustained over activation of NMDAR in those brain regions. The argument gets support from the increased expressions of NR2A and NR1 sub units of the NMDAR consistent with significantly increased levels of nNOS and NO in the brain of the HA rats (unpublished data).

Alternatively, irrespective of the nature of ROS inducer, the brain cells operate an effective system of antioxidant enzymes to regulate ROS metabolism [8]. In the present context, therefore, we studied the profile of the main antioxidant enzymes vis a vis oxidative stress markers in the cortex and cerebellum of the ALF rats. Brain is structurally rich in polyunsaturated fatty acids and has high oxygen demand. This makes it the most vulnerable organ to undergo oxidative damage during un-physiological challenges. Therefore, MDA level, the first stable product of lipid peroxidation, and the protein carbonyl contents, resulting from the ROS mediated protein oxidation; serve as immediate indicators for the oxidative damage produced by ROS in the brain cells [15]. We observed significant increase in the levels of both these oxidative markers in cerebral cortex and cerebellum of the ALF rats (Table-3). Such a condition might indicate for diminished antioxidant mechanisms to counterbalance deleterious effects of ROS in brain of the ALF rats. Indeed, it was found to be true in light of significant and concordant decline in the levels of all the antioxidant enzymes in cerebral cortex and cerebellum of the ALF rats (Fig-3, 4, and 5).

SOD is the committed enzyme of the antioxidant pathway and catalyzes dismutation of O_2_^•-^ to produce H_2_O_2_ and O_2_. H_2_O_2_ is then metabolized by either catalase and/or GPx to prevent ROS induced cellular/molecular damage [36]. Interestingly, SOD level showed an increase in cerebral cortex but with a significant decline in cerebellum of the ALF rats (Fig-3). Since, the pattern corroborated well with a similar type of differential SOD levels observed in case of the fulminate hepatic rats [16] and that it has re-affirmed our earlier report describing opposite trend of SOD changes in cerebral cortex and cerebellum of the rats with a milder level of ammonia in brain [7], it is argued that both these brain regions respond differentially with respect to the changes in the antioxidant enzymes during hyperammonemic exposure. Moreover, significant decline in the levels of catalase and GPx, the two H_2_O_2_ degrading enzymes in both the brain regions of the ALF rats (Fig. 4, 5), provide important metabolic relevance for the differential pattern shown by SOD. The increased levels of both, the O_2_^•-^ and H_2_O_2_, are considered equally effective to cause oxidative damage at cellular level [15]. In case of cerebral cortex, the combination of increased SOD and declined catalase and Gpx levels may lead into accumulation of H_2_O_2_ in this brain region of the ALF rats. Similarly, the combination of declined SOD-catalase and GPx activity is likely to increase the O_2_^•-^ load in both the brain regions. This could be the reason for observing significantly increased levels of lipid peroxidation and carbonylated protein (Table-3) in these brain regions of the ALF rats. This explanation gets support from a recent report from our lab describing the importance of concordant changes in the antioxidant enzymes to normalize oxidative stress in the brain slices exposed with even moderate level ammonia [31].

To verify further the role of brain antioxidant mechanisms in inducing oxidative stress during ALF, regulation of GSH turnover was also monitored. GSH is a tripeptide that plays important roles in maintaining reducing equivalents in the cells facing oxidative challenges [37]. In mammalian cells, the turnover of GSH is mainly regulated by the synchronized activities of GPx (GSH utilizing enzyme) and GR (GSH recycling enzyme). In cerebellum of the ALF rats, we observed significantly decreased level of GPx (Fig. 5) but no change in GR activity (Figs. 6). Such a pattern of GPx vs GR is thus, likely to prevent GSH depletion in this brain region. Indeed, endogenous level of GSH was found to be stable in cerebellum of the ALF rats (Table-3). Moreover, cerebral cortex also showed GPx-GR profile similar to that of the cerebellum (Fig-6) but significantly increased level of GSH (Table-3). Such a mismatch could arise due to GSH supply from the additional biochemical routes in this brain region. The HA induced increased activity of gamma glutamyl-cystein synthetase, a GSH producing enzyme, could be one of the alternatives in the present context, as this mechanism has been attributed to produce extra GSH in the brain cells exposed with the hyperammonemia [38].

Taking together, it is evident that though cerebral cortex and cerebellum enzymatically adapt to resist GSH depletion, yet they become vulnerable to oxidative damage during ALF condition mainly due to decline in SOD-catalase/GPx activity.

In conclusion, it is evident that GS-GA cycle gets activated in cerebral cortex and cerebellum of the ALF rats resulting into efficient ammonia metabolism and increased glutamate turnover in these brain regions. This suggests activation of a biochemical mechanism to prevent ammonia accumulation in the brain during ALF. Moreover, ALF induced oxidative damage in both these brain regions could not be prevented mainly due to concordant decline in the levels of all the antioxidant enzymes. Thus, though a direct correlation between glutamate level and oxidative stress could not be established, oxidative stress, as an independent factor, is evident to play important roles in the pathogenesis of acute type HE. As such this paper provides a biochemical basis to target antioxidant mechanisms for therapeutic management of HE.
